# Comparative Efficacy and Mechanisms of a Single-Session Pain Psychology Class in Chronic Low Back Pain: Study Protocol for a Randomized Controlled Trial

**DOI:** 10.1186/s13063-018-2537-3

**Published:** 2018-03-06

**Authors:** Beth D. Darnall, Maisa S. Ziadni, Anuradha Roy, Ming-Chih Kao, John A. Sturgeon, Karon F. Cook, Kate Lorig, John W. Burns, Sean C. Mackey

**Affiliations:** 1 Department of Anesthesiology, Perioperative and Pain Medicine, Division of Pain Medicine, Stanford University School of Medicine, Stanford University, Stanford Systems Neuroscience and Pain Lab, 1070 Arastradero Road, Suite 200, MC 5596, Palo Alto, CA 94304 USA; 2 Feinberg School of Medicine, Northwestern University, Evanston, IL USA; 3 Department of Behavioral Sciences, Rush University Medical Center, Chicago, IL USA

**Keywords:** Back pain, Pain catastrophizing, Cognitive behavioral therapy, Chronic pain, Psychology, Treatment

## Abstract

**Background:**

The Institute of Medicine (IOM) reported that chronic pain affects about 100 million U.S. adults, with chronic low back pain (CLBP) cited as the most prevalent type. Pain catastrophizing is a psychological construct shown to predict the development and trajectory of chronic pain and patient response to pain treatments. While effective treatment for pain catastrophizing typically includes eight-session groups of cognitive behavioral therapy (CBT), a single-session targeted treatment class yielded promising results which, if replicated and extended, could prove to efficiently and cost-effectively reduce pain catastrophizing. In this trial, we seek to determine the comparative efficacy of this novel single-session pain catastrophizing class to an eight-session course of pain CBT and a single-session back pain health education class. We will also explore the psychosocial mechanisms and outcomes of pain catastrophizing treatment.

**Methods:**

In this trial we will randomize 231 individuals with CLBP to one of three treatment arms: (1) pain-CBT (eight weekly 2-h group sessions with home exercises and readings); (2) a single 2-h pain catastrophizing class; or (3) a single 2-h back pain health education class (active control). For the primary outcome of pain catastrophizing, the trial is designed as a non-inferiority test between pain-CBT and the single-session pain catastrophizing class, and as a superiority test between the single-session pain catastrophizing class and the health education class. Team researchers masked to treatment assignment will assess outcomes up to six months post treatment.

**Discussion:**

If the single-session targeted pain catastrophizing class is found to be an effective treatment for patients with CLBP, this low cost and low burden treatment could dismantle many of the current barriers and burdens of effective pain care. Further, elucidation of the mechanisms of pain catastrophizing treatments will facilitate future research on the topic as well as further development and refinement of treatments.

**Trial registration:**

ClinicalTrials.gov, NCT03167086. Registered on 22 May 2017.

**Electronic supplementary material:**

The online version of this article (10.1186/s13063-018-2537-3) contains supplementary material, which is available to authorized users.

## Background

The Institute of Medicine (IOM) recently reported that chronic pain affects about 100 million U.S. adults and costs the nation $635 billion annually in medical costs and lost productivity [1]. Chronic low back pain (CLBP) is cited as the most prevalent type of chronic pain [1] and rates continue to rise despite increased utilization of treatments such as surgery and pharmacology. For instance, in North Carolina the prevalence of CLBP rose from 3.9% in 1992 to 10.2% in 2006, along with disability and healthcare costs [2]. Hoy et al. reported a low back pain one-year period prevalence of 38% [3]. This demonstrates the growing need for effective interventions to prevent CLBP and better treat it once established. Multiple studies suggest that the greatest predictor for the development and progression of CLBP is pain catastrophizing (PC) [4]. PC is a cognitive-emotional response pattern involving negative expectation and appraisal about actual or anticipated pain and includes feelings of helplessness about pain [5]. Consistently across studies, PC is a primary predictor for the onset and worsening of CLBP, even among all surgical and clinical variables [4, 6, 7]. While PC is effectively treated with pain-cognitive behavioral therapy (CBT), access to care may be limited by several barriers, including the need for physician referral, poor reimbursement or no insurance, co-payment costs, and time and costs associated with the 6–10 group or individual pain-CBT treatment sessions. This study addresses the critical problem of poor access to PC treatment among patients with CLBP.

A single-session targeted class was recently shown to reduce pain catastrophizing at four-week follow-up in a cohort of 57 mixed etiology chronic pain patients receiving treatment at a tertiary referral, multidisciplinary chronic pain clinic [8]. The effectiveness of the class was found to be equivocal for patients with co-morbid anxiety and depression compared to those without these diagnoses. The results of this pilot study, while preliminary, are particularly promising because the intervention is efficiently delivered in a single 2-h class. If proven effective in a larger, randomized and controlled study, many of the barriers associated with traditional, multi-session pain-CBT would be eliminated for those seeking focal catastrophizing treatment.

### Specific aims

Our two specific aims and their corresponding hypotheses are outlined below.

1. To implement a comparative efficacy trial of a single-session pain psychology (SPP) class that targets PC with the standard of care, eight-session pain-CBT (8-CBT), and a single-session back pain health education class (Control).

- *Hypothesis 1a* (Superiority): The SPP class will be superior to Control for Trait PC reduction.
- *Hypothesis 1b* (Assay sensitivity): 8-CBT will be superior to Control for Trait PC reduction.
- *Hypothesis 1c* (Non-inferiority): The SPP class will be non-inferior to 8-CBT for Trait PC reduction.
- *Hypothesis 1d* (Global improvement): The SPP class will be superior to Control and non-inferior to 8-CBT for longitudinal changes in PROMIS pain intensity and behavior, fatigue, and sleep disturbance.
- *Hypothesis 1e* (Improvement in objective measurement of function): Reductions in PC in the SPP class is associated with improvements in objective physical function as measured by actigraphy.

2. To characterize the mechanistic influence of Daily PC on future pain, function, and Trait PC.

- *Hypothesis 2a* (Level-1 PC effect): Daily PC will predict same-day and next-day levels of pain and activity.
- *Hypothesis 2b* (SPP/CBT moderation of Level-1 PC lagged effect): The relationships between Daily PC and same-day and next-day pain and activity are reduced by SPP and CBT interventions compared to the health education class.
- *Hypothesis 2c* (Level-2 SPP effect): Daily PC mean changes (baseline to one-month post treatment) will predict mean change in pain, activity, sleep, and Trait PC in the SPP and 8-CBT groups at months 1–3.
- *Hypothesis 2d* (Level-1 SPP/CBT skills use lagged effect): Previous-day and same-day use of SPP/CBT skills will predict next day improvement in Daily PC.

## Methods/Design

### Overview

**Fig. 1:**
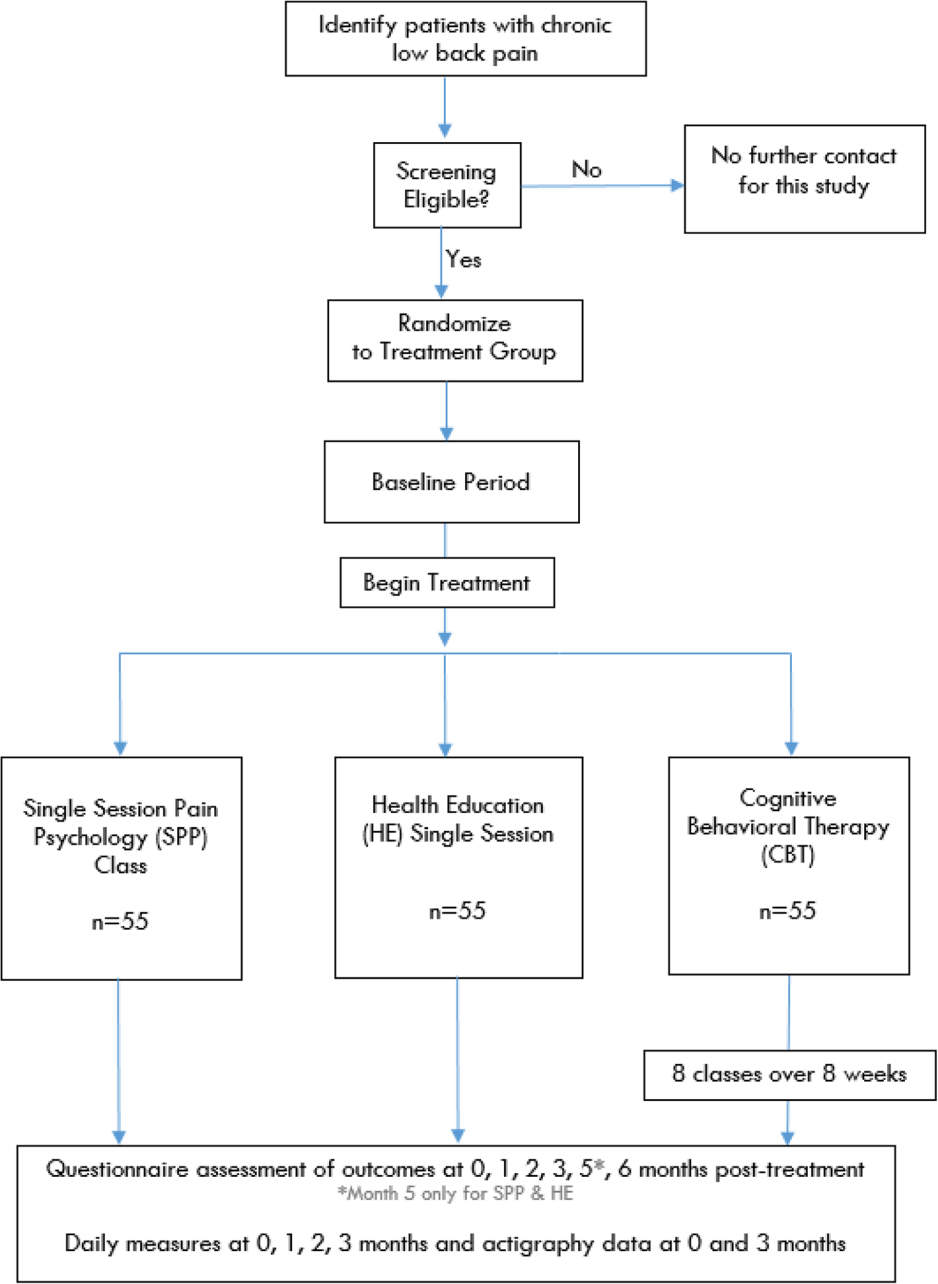
Flowchart of the trial protocol

**Fig. 2:**
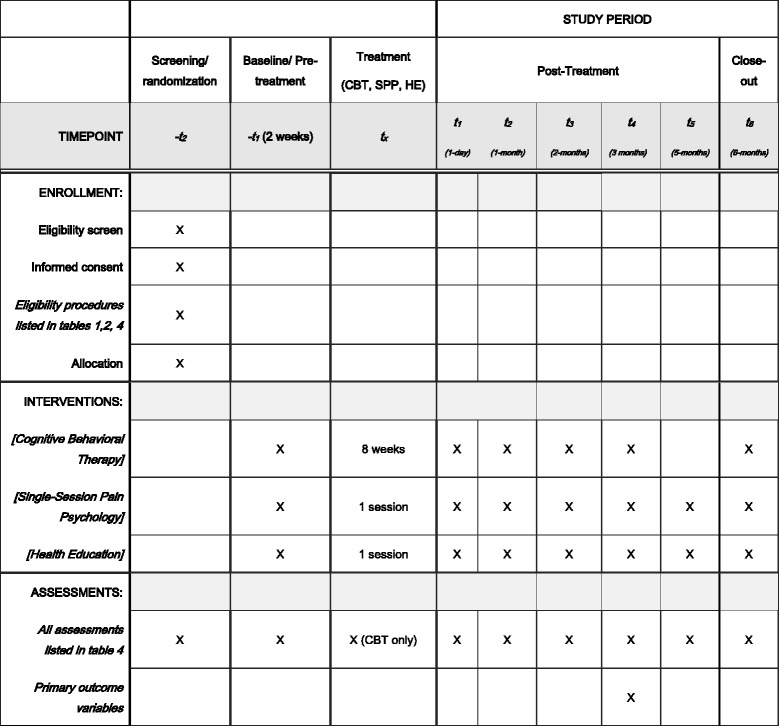
The schedule of enrollment, interventions, and assessments

We are conducting a randomized clinical trial in which individuals with CLBP are randomly assigned to eight-session CBT group, single-session pain psychology class, and a single-session back pain health education class (active control; “Control”) (Figs. 1 and 2). Participants will be followed for 7–9 months after randomization, depending on the treatment group assignment. Participants will be assessed at screening visit, two-week baseline period, pre-treatment visit, post treatment, and up to five post-treatment follow-ups over six months. During their pre-treatment visit, participants randomized to the single-session pain psychology class receive an audio app on their mobile phone or portable electronic device. Team statisticians blinded to participant treatment assignment will assess outcomes immediately following treatment, and after one, two, three, and five months (for SPP and health education [HE] only), and at six months post treatment. The primary outcome is Trait PC at three months post treatment. Secondary outcomes will include Trait PC at six months post treatment and PROMIS measures at three month post treatment in each of the core outcome domains for chronic pain clinical trials identified by the Initiative on Methods, Measurement, and Pain Assessment in Clinical Trials (IMMPACT) consensus panel [9] and the NIH Task Force on Research Standards for Chronic Low Back Pain [10]. These include pain intensity, physical functioning, and emotional functioning. Additionally, secondary outcomes will include actigraphy data for function and sleep.

The protocol for this trial has been approved by the Stanford Institutional Review Board (IRB). All participants will be required to give their informed consent at screening and before enrollment in the study. Consent will be obtained by trained study team members.

### Study sample and setting

Participants for this trial will be recruited from social media marketing, the Stanford Systems Neuroscience and Pain Lab database, and local advertisements in clinics and in the community. All advertisements will direct interested individuals to complete an online screening form to assess for their eligibility. The study will enroll 231 adults (age 18–70 years) with axial low back pain without radicular symptoms who meet study criteria (Table 1). This sample size accounts for expected attrition. Eligibility will be assessed by the research staff.

### Inclusion and exclusion criteria

Tables 1 and 2 list the inclusion and exclusion criteria, respectively, as well as the rationale for each criterion and the sources where each criterion will be accessed. Additionally, we require that participants be willing and available to participate in the full treatment session to which they are assigned (ranging from a single treatment session to eight treatment sessions over eight weeks). We also require that participants are able to complete daily measures and actigraphy and be able to respond to the post-treatment (0, 1, 2, 3, 5, 6 months) follow-up questionnaires.

**Table 1:** Inclusion criteria

Inclusion criteria	Rationale	Sources
Axial low back pain without radicular symptoms	Study restricted to low back pain	A, TS/S
Pain duration ≥ 6 months	As per recent NIH Task Force on Research Standards for Chronic Low Back Pain	A, TS/S
Average pain intensity ≥ 4/10 for the past month at screening visit	Significant level of back pain to treat and to detect improvement	A, TS/S
English fluency		A, TS/S
Men and women aged 18–70 years		A, TS/S
PCS score ≥ 20	Significant level for PC [4]	TS/S

*A* automated data gathered from REDCap Surveys, *TS* telephone screening, *S* screening visit, *PCS* Pain Catastrophizing Scale

**Table 2:** Exclusion criteria

Exclusion criteria	Rationale	Sources
Gross cognitive impairment	Condition which would make it difficult for a person to consent and partake in the study	TS/S
Active suicidal ideation or severe depression		S
Previous attendance in the active treatment groups	Possible bias due to prior exposure to treatment groups	TS/S
Participating in any interventional research study or completed participation in the last 2 months; enrollment in an observational study is acceptable	Treatment interference	TS/S
Current substance abuse		S
Clear likelihood to disrupt fellow class participants (e.g. personality disorder) at the discretion of the study team		TS/S
Any radicular symptoms	Study restricted to low back pain	TS/S
Ongoing legal or disability claim, Worker’s Comp (permanent and stationary disability not exclusionary)		TS/S
Currently pregnant or planning to become pregnant		TS/S
Average pain intensity < 4/10 for the past month at screening visit	Back pain too moderate to treat and to detect improvement	A, TS/S
Disorders indicated by the MINI self-report questionnaire will be characterized and participants may be excluded at the discretion of the researcher	Condition which would make it difficult for a person to partake in treatments (e.g. social anxiety disorder would inhibit a person’s ability to fully participate in group treatment)	S

*A* automated data gathered from REDCap Surveys, *B* baseline period, *TS* telephone screening, *S* screening

### Recruitment procedures

Because the study intervention involves classes, we are recruiting participants in cohorts consisting of 7–12 participants per class cohort (minimum of four participants, maximum of 20 participants per cohort) for all three study arms.

Interested individuals will be screened over the phone or will complete an online screening form. Potentially eligible individuals will be invited to an on-site screening visit where eligibility will be determined. The Mini International Interview (MINI 7.0) will be administered to exclude for suicidality and current substance abuse, and to characterize other psychiatric disorders. Eligible individuals will complete the consenting and enrollment procedures. Participants are randomized following eligibility confirmation and informed consent procedures.

### Randomization

Enrolled participants will be randomized to one of three treatment arms: pain psychology class (SPP), eight-week CBT (pain-CBT), and back pain health education (HE). An automated program in REDCap will randomly assign a participant to a treatment arm when enrolled and will ensure blinded randomization and equal numbers in all three treatment arms at the end of data collection.

### Blinding

Participants will not be blinded to the intervention they are randomized to. Treatment providers and study coordinators will not and cannot be blinded to treatment allocation. The study coordinator will be responsible for handling the randomization process through REDCap; however, they are blinded to the randomization scheme. This study coordinator will coordinate delivery of intervention; therefore, will not have access to data, data monitoring, or analysis of data. An alternative research team member will have access to the data and will be responsible for the data monitoring. The principal investigators, co-investigators, and statisticians will remain blinded through the data collection phase. The team will have access to the final un-identified dataset.

### Study treatments

Both the SPP and HE group sessions consist of one 2-h class. The SPP treatment includes assigned home activities. The pain-CBT group treatment consists of eight weekly 2-h sessions which will also be supplemented by home activities.

#### Cognitive-behavioral therapy (CBT)

CBT for chronic pain is well-documented in the literature and has been found to be modestly to moderately effective in improving chronic pain [11–14]. For this study, we will be using the manualized CBT treatment manual utilized by Cherkin et al. [15, 16].

The CBT intervention (Table 3) will consist of eight weekly 2-h sessions that will provide: (1) education about the role of maladaptive thoughts and beliefs common in people with chronic pain; and (2) instruction and practice in identifying and challenging negative thoughts, the use of thought-stopping techniques, the use of positive self-coping statements and goal-setting, relaxation techniques, and coping with pain flares. The intervention will also include education about activity pacing, relapse prevention, and maintenance of gains. During each session, participants will complete a personal action plan for activities to be completed between sessions. These plans will be used as logs for setting specific home practice goals to be reviewed at the next week’s session. Participants will be given a copy of *The Pain Survival Guide: How to reclaim your life* © 2005 by Turk & Winter [17] for optional reading. The threshold for pain-CBT completion is attendance at five of the eight classes.

**Table 3:** Content of each group treatment (CBT, SPP, and HE) session

Session	CBT		SPP	HE
1	Welcome and Introduction; CBT rationale and evidence; Pain physiology; Relaxation rationale; Importance of home practice	Single session	Single 2-h session, Didactic and Skills Acquisition *Didactic*: Learn about mind– body science related to pain and PC; learn to identify catastrophizing in the moment; and how to self-treat it. *Skills Acquisition*: Developing a plan to apply the learned skills to decrease physiological hyperarousal – diaphragmatic breathing and progressive muscle relaxation – within the context of PC. Improve the regulation of cognition and emotion, including PC reframing and thought restructuring. Developing a plan for implementing these skills in daily life. Identifying typical PC thoughts and practice writing out their reframes. Participants leave with the following tangibles: (1) self-written, self-crafted, personalized PC cessation plan; (2) 20-min relaxation response audio CD/app; and (3) printed copy of the SPP content to access as needed in their PC cessation plan.	Single 2-h session, Class delivered via a PowerPoint presentation to groups of participants. *Content*: Overview of back pain, including common sources and red flag symptoms. Also covers topics including managing a pain flare-up, working with healthcare professionals, evaluating treatments and making informed treatment decisions, and achieving and maintaining a healthy weight through good nutrition and exercise.
	*Relaxation*: Intro to diaphragmatic breathing			
2	Goal setting, activation, and pacing (SMART, rest-activity cycle, etc.); Red flags; Coping with flare-ups and creating a flare-up plan			
	*Relaxation*: 7-muscle group progressive muscle (PMR) relaxation			
3	Role of thoughts and feelings in pain; Intro to CBT and terms; Intro to 3-column thought record			
	*Relaxation*: 4-muscle group PMR; fitting diaphragmatic breathing into daily life			
4	Evaluating and generating alternate thoughts; Intro to evidence gathering; Intro to 4-column thought record			
	*Relaxation*: 4-muscle group PMR; no tension			
5	More on evidence gathering and alternate thoughts (more detail); Working with thoughts review			
	*Relaxation*: Body scan			
6	Thought records review			
	*Relaxation*: Walking body scan			
7	Review of skills; Trouble-shooting re: thought records; Pain and mood; Pain core beliefs			
8	Review of skills; “Signs” not using skills: Creating a plan for maintaining gains and dealing with setbacks; Termination and wrap-up			
	*Relaxation*: Guided imagery			

*CBT* cognitive behavioral therapy, *SPP* single-session pain psychology class, *HE* health education, *SMART* specific, measurable, assignable, realistic, time-related, *PMR* progressive muscle relaxation, *PC* pain catastrophizing

#### Single-session pain catastrophizing class

The pain catastrophizing class is a unique intervention that specifically treats PC. Our pilot data show that the PC class significantly reduced PC one month post treatment, even in patients with co-morbid depression and anxiety [8]. For this study, SPP will be delivered via a PowerPoint presentation to groups of participants in a single 2-h class. SPP has two main components: didactics and skills acquisition (summarized in Table 3). Didactic content includes mind–body science as it relates to pain and PC. Participants learn how to identify catastrophizing in the moment and how to self-treat it. Participants self-tailor the information relayed during the class by developing their own comprehensive self-treatment plan to stop and prevent catastrophizing. Participants leave the class with the following tangibles: (1) their self-written personalized PC cessation plan; (2) an app with a 20-min guided relaxation response audio file; and (3) a printed copy of the SPP class content. Cohort effects are expected to be minimal due to the single-session nature of the class, the fact that the class content is highly structured and manualized, and because participant interaction is relatively minimal.

#### Back health education

The HE class will be delivered via a PowerPoint presentation to groups of participants in a single session lasting approximately 2 h. The HE class will provide the group with an overview of back pain, including common sources and red flag symptoms. Didactics will also cover topics including working with healthcare professionals, evaluating treatments and making informed treatment decisions, and achieving and maintaining a healthy weight through good nutrition and exercise. The back HE class serves to control for the non-specific effects of: (1) receiving a “treatment;” (2) participating in a research study; and (3) providing daily ratings for PC and pain. The 2-h HE group will match SPP on four important factors: duration; structure; format; and site [18].

### Class sites

All treatment sessions will occur at approved clinical or research sites within the Stanford University School of Medicine and Stanford HealthCare.

### Instructors

For the active psychological treatment groups, all instructors will be doctoral level clinical psychologists trained in the treatment of chronic pain. The HE class will be expert-led by experienced health educators or chronic pain professionals (e.g. chronic pain physician assistant). The psychologist instructors for the CBT and the SPP will be mutually exclusive to enhance content distinction for the purposes of this comparative trial. Instructor effects will be examined analytically.

### Training and monitoring of instructors

All CBT and SPP instructors will be trained in the study protocol for the respective interventions. For CBT, treatment fidelity checklists highlighting the essential components of each session were created based on those developed by Cherkin et al. (2014). A member of the research staff will be trained on the CBT manualized protocol and will assess treatment fidelity using session data and the fidelity checklist. He/she will analyze and rate a random sample of 20% of the recorded sessions for fidelity to the manualized CBT protocol. The study’s principal investigators will review the ratings to confirm fidelity. A research team member will serve as fidelity rater for the SPP class. In structure and format, SPP is optimized for treatment fidelity because it has standardized content and standardized handouts and materials. The research coordinator will directly observe the first three SPP classes and up to three SPP classes at random to ensure treatment fidelity. In addition, the research coordinator will complete the fidelity sheets for the HE class. Fidelity is built into the HE class as well with the use of PowerPoint that makes the curriculum very structured and difficult to deviate from.

### Measures

The M.I.N.I. International Neuropsychiatric Interview (M.I.N.I. 7.0) will be administered to exclude for suicidality and current substance abuse and to characterize other psychiatric disorders. We will assess a variety of baseline characteristics, including sociodemographic characteristics and medical history and medications, in addition to treatment expectations. The Beck Depression Inventory-II (BDI-II) and, if necessary, the Structured Clinical Interview for DSM-5 (SCID-5) will be administered to assess the severity of depressive symptoms (Table 4).

**Table 4:** Baseline and follow-up measures

Measurement domain/ Name of measure	Brief measure description	Screening	Baseline period	Pre-treatment	Mid-treatment	Post-tx month 0	Post-tx months 1, 2 3, 5, 6
Demographics	Age, gender, race, ethnicity, handedness, education level, household income, employment	x					
Medical history and medications	Height, weight, smoking, back pain etiology, pain duration, pain intensity, other pain conditions, pain treatments, psychological conditions, medications	x					
Mini International Neuropsychiatric Interview (MINI) [36]	A MINI screen will be administered for two reasons: (1) for exclusionary criteria assessment for suicidality and current substance abuse; (2) to characterize other psychiatric disorders. Positive items will be followed by administering relevant modules of the Structured Clinical Interview for DSM (SCID) by a trained member of the study team	x					
Beck Depression Inventory-II (BDI-II) [37]	BDI-II, the updated version of the 21 items questionnaire which assesses the intensity of depression in patients. It is the most widely used instrument for detecting depression (http://www.pearsonclinical.com/psychology/products/100000159/beck-depression-inventoryii-bdi-ii.html#tab-details)	x					
Structured Clinical Interview for DSM-5 (SCID-5) [38]	A semi-structured interview guide for making DSM-5 diagnoses. It is administered by a clinician or trained mental health professional that is familiar with the DSM-5 classification and diagnostic criteria (https://www.appi.org/products/structured-clinical-interview-for-dsm-5-scid-5)	x					
Chronic Pain Acceptance Questionnaire (CPAQ-8) [39]	Eight question version CPAQ has been designed to measure acceptance of pain. The acceptance of chronic pain is thought to reduce unsuccessful attempts to avoid or control pain and thus focus on engaging in valued activities and pursuing meaningful goals			x	x	x	x
West Haven-Yale Multidimensional Pain Inventory (WHY-MPI) [40]	West Haven-Yale MPI is designed to provide a brief, psychometric assessment of important components of the chronic pain experience. In particular, this study will be looking at how a significant other responds to a participant when they are in pain			x	x	x	x
Body map	Interactive map of male/female body to select regions that experience pain			x	x	x	x
Back pain bothersomeness	Single item measure of back pain bothersomeness in the past week from not at all bothersome to extremely bothersome			x	x	x	x
Satisfaction with Life Scale [41]	A 5-item scale designed to measure global cognitive judgments of one’s life satisfaction. Participants indicate how much they agree or disagree with each of the 5 items using a 7-point scale that ranges from 7 strongly agree to 1 strongly disagree			x	x	x	x
Perceived Stress Scale [42]	10 items on a 5-point scale from never to very often measuring aspects of perceived stress in the past month. Items were designed to measure how unpredictable, uncontrollable, and overloaded respondents perceive their life to be			x	x	x	x
Childhood Trauma Questionnaire [43]	The self-report measure includes 28 items that measure 5 types of maltreatment: emotional, physical, and sexual abuse; and emotional and physical neglect. Items are measured on a 5-point Likert scale with responses ranging from never true to often true			x			
Positive and Negative Affect Schedule [44]	10 positive affect descriptors and 10 negative affect descriptors are measured for the resent moment on a 5-point scale ranging from very slightly or not at all to extremely			x	x	x	x
Patient’s Global Impression of Change [45]	One item measure of status change since start of treatment and one item measure of side effects					x	x
Working Alliance Inventory [46]	12 items on 7-point scale from never to always measuring how the participant feels about the treatment group instructor				x	x	
Treatment expectancies [47]	Stanford Expectations of Treatment Scale, a 6-item tool our group developed and validated at SNAPL, will be used to assess participant expectations of treatment			x			
Mid Credibility Expectancy Questionnaire [48]	4 items assessing patient impressions of the treatment midway through treatment participation				x		
Post Credibility Expectancy Questionnaire [48]	4 items assessing patient impressions of the treatment post-treatment participation					x	
Tx satisfaction, utility, and knowledge	7 items assess participant satisfaction and perceived utility of treatment on a 7-point rating scale. For the SPP group, 5 items will assess knowledge acquired regarding PC and self-treatment					x	
Treatment and life event changes	Assesses new treatments, major lifestyle changes, major or adverse life events (negative and positive), and new injuries at different time points throughout the study			x	x	x	x
Skills use	Single item measure assessing frequency of skills use learned in class over the past month from not at all to several times per day						x
Primary treatment outcome measures							
Pain Catastrophizing Scale (PCS) [5]	13-item scale assesses severity of Trait PC tendencies on a 5-point scale (0 = “not at all” to 4 = “all the time”); sum scores are in the range of 0–52.The PCS has 3 factors (helplessness, magnification, rumination) and has good psychometrics [5]. The PCS total score at 3 months post treatment is our primary endpoint (Trait PC)	x		x	x	x	x
NIH PROMIS measures [49]	NIH PROMIS measures have been successfully applied in pain research [49–52] and will be used to assess multiple variables of interest, including Pain Intensity, Pain Interference, Pain Behavior, Function, Depression, Anxiety, Sleep Disturbance, Sleep Interference, Anger, and Fatigue. Computerized automated testing [53] technology will be used to minimize participant burden. Participants also complete PROMIS Global Health at the same time points.			x	x	x	x
Pain Self-Efficacy Questionnaire (PSEQ) [28]	10-item instrument measures self-confidence to manage pain and engage in life activities despite pain [24, 26–29, 54]. Pain self-efficacy be used as a mediating / moderating / process variable			x	x	x	x
Daily Measures / ESM (2 week data waves)							
Daily PCS	The 5-item Daily PCS will be administered daily		→		→		0–3 mos. →
Daily pain, mood, and function questions	In a single question for each theme, for the past 24 h, average back pain, highest level of back pain, pain interference, stress, positive emotions, negative emotions, and satisfaction with life as assessed		→		→		0–3 mos →
Skills use *(only for SPP & CBT)*	SPP group will answer 3 questions assess use of cognitive, behavioral, and psychophysiological techniques over 24 h from 0 times to 5+ times. The pain-CBT group will complete these questions daily after the relevant material is covered in class						0–3 mos →
Objective data							
Sleep and activity	Actigraphy devices will quantify 24 h sleep and activity variables including: total sleep time and efficiency, energy expenditure, MET rates, steps taken, physical activity intensity		→		→		0 and 3 mos →
Guided relaxation (per participant discretion/ goal settings; continuous data)	An app will timestamp each time an SPP participant accesses the “SPP Relaxation Resource” on their device, thus providing an objective measure of skills use						0–3 mos *(SPP)* →

*MINI* Mini International Neuropsychiatric Interview, *WHYMPI/MPI* West Haven–Yale Multidimensional Pain Inventory

#### Baseline period

Within 30 days of starting treatment, participants will complete a two-week baseline period of daily pain measures. Participants will also wear the actigraphy unit to collect activity/rest/sleep measurements during this period.

#### Pre-treatment assessment

Three days before treatment, participants will be administered an online pre-treatment assessment of pain symptoms, emotional functioning, PCS, and general health and wellbeing.

#### Mid-treatment assessment

At four weeks, there will be a mid-treatment assessment for the pain-CBT group only. Daily pain measures and actigraphy data will also be collected from weeks 3–5 for CBT participants.

#### Post-treatment assessment

We will administer a core set of outcomes measures immediately following treatment, and at one, two, three, five (for SPP and HE only), and six months post treatment. Two-week daily measures will be collected immediately post treatment and at one, two, and three months post treatment. Actigraphy data will also be collected immediately post treatment and at three months post treatment. The primary study endpoint is three months.

Participants may receive up to $300 for study completion.

##### Primary outcome measures

Our primary outcome measure is Trait PC at three months post treatment. Trait PC will be assessed using the sum score for the PCS [2, 19], a 13-item scale used to assess severity of Trait PC tendencies on a 5-point scale (0 = “not at all” to 4 = “all the time”). The PCS has three factors (helplessness, magnification, rumination) and has good psychometrics [20].

NIH PROMIS [21] will be administered to assess multiple variables of interest, including Pain Intensity, Pain Interference, Pain Behavior, Function, Depression, Anxiety, Sleep Disturbance, Sleep Interference, Anger, Global Health, and Fatigue. NIH PROMIS measures have been successfully applied in pain research [14, 22–24].

Participant self-confidence regarding their ability to participate in various life activities despite their pain will be assessed using the Pain Self-Efficacy Questionnaire (PSEQ) [25], a ten-item self-report instrument [23, 24, 26–29].

##### Daily measures

All participants will complete daily measures for a two-week period at each of the following time points: pre-treatment baseline; mid-treatment (for eight-week CBT); immediately after treatment; and at one, two, and three months post treatment. Daily measures will include the five-item Daily PCS, daily pain, mood, and function questions. In a single question for each theme, for the past 24 h, average back pain, highest level of back pain, pain interference, stress, positive emotions, negative emotions, and satisfaction with life are assessed. Skills use will be assessed by asking participants (SPP and CBT) to respond to three questions measuring frequency of use of cognitive, behavioral, and psychophysiological techniques over 24 h from 0 times to 5+ times. The pain-CBT group will complete these questions daily after the relevant material is covered in class.

##### Objective data

Sleep and Activity will be assessed with actigraphy devices that quantify 24-h sleep and activity variables including: total sleep time and efficiency; energy expenditure; MET rates; steps taken; and physical activity intensity. A guided relaxation app will timestamp each time an SPP participant accesses the “SPP Relaxation Resource” on their device, thus providing an objective measure of skills use.

### Data collection, quality control, and confidentiality

All questionnaires will be completed by participants in a REDCap database. Questionnaires collected on paper (due to unforeseen circumstances) will be stored as source data and a member of the study team will enter the data into the REDCap database. All staff will receive training on completing case report forms (CRFs) appropriately, reviewing CRFs for completeness, and maintaining participant confidentiality. We will collect information at every stage of recruitment, randomization, and treatment so that we can report patient flow according to the CONSORT (Consolidated Standards of Reporting Trials) guidelines [30].

### Protection of human participants and assessment of safety

#### Protection of human participants

The Stanford University Institutional Review Board (IRB) approved this study.

#### Safety monitoring

This trial will be monitored for safety by an independent Data and Safety Monitoring Board (DSMB) composed of a clinical psychologist, a biostatistician, and a professor of psychiatry and behavioral medicine with expertise in chronic pain.

#### Adverse experiences

Adverse events (AEs) will be reported to the National Center for Complementary and Integrative Health (NCCIH), DSMB, and Stanford Institutional Review Board (IRB) annually. Serious adverse events (SAEs) that are determined to be related to the study will be reported to the IRB by filing a report on the Stanford IRB website. A copy of this report will be sent to the NCCIH officer. Unexpected fatal or life-threatening AEs related to the intervention will be reported to the NCCIH Program Officer within seven days. Other serious, unexpected, and related AEs will be reported to the NCCIH Program Official within 15 days and to the Stanford IRB within ten business days. Anticipated or unrelated SAEs will be handled in a less urgent manner but will be reported to the Independent Monitor(s), Stanford IRB, and NCCIH in accordance with their requirements. In the annual AE summary, the Independent Monitor(s) Report will state that they have reviewed all AE reports.

#### Stopping rules

The trial will be stopped if the DSMB determines that there is a risk of SAEs in one or more of the treatment arms. In this case, the DSMB can decide if one of the treatment arms needed to be terminated.

### Statistical issues

#### Sample size and detectable differences

Our sample size was chosen to ensure adequate power to detect significant differences between each of the two treatment groups and the HE, as well as power to detect a statistically significant difference between the two treatment groups. The project will enroll 231 adults (ages 18–70 years) who meet criteria for axial low back pain without radicular symptoms.

To compare the mean difference in the SPP group against the HE group using a two-sample t-test, we conservatively plan to enroll 231 participants and have 165 completers (55 per group). The proposed sample size accounts for attrition at 25% for single session treatments and 35% for eight-session CBT. With this, we achieve 90% power to reject the null hypothesis of equal means when the population mean difference is 5 (63% of that seen in the pain-CBT literature; 45% of that seen in Preliminary Study 1) and 80% power to reject the null when the population mean difference is 4.3 (54% of that seen in the pain-CBT literature; 40% of that seen in Preliminary Study 1), with standard deviation (SD) for both groups of 8, α = 0.05, using a two-sided, two-sample equal-variance t-test.

For our primary analyses examining PCS scores at three months, the mean difference in the pain-CBT group is compared against the HE group using a two-sample *t*-test. The mean effect of pain-CBT on PCS is about 8 in the literature [14, 31]. Group sample sizes of 55 each in the pain-CBT and HE groups achieve 90% power to reject the null hypothesis of equal means when the population mean difference is 5 (63% of that seen in literature) and 80% power to reject the null when the population mean difference is 4.3 (53% of that seen in literature), with a SD for both groups of 8 and with a significance level (α) of 0.05 using a two-sided, two-sample equal-variance *t*-test.

For assessing whether SPP provides durable and substantial reduction in PC non-inferior to pain-CBT, a group sample size of 55 in each group achieve 80% power with margin of non-inferiority 4.3. The true difference between the means is assumed to be 0, the significance level (alpha) of the test is 0.025, and the population SD is 8 in both groups. Given the effect of eight-week CBT on PCS is about 8 [14, 31], this non-inferiority margin would imply the single-session SPP retains half of the effect of the eight-session pain-CBT on PCS, a clinically well-accepted level of superiority retention [32–34].

The association between changes in PC provided by SPP and objective physical function will be measured with correlation coefficient between changes from baseline to primary endpoint in PC and in each of the actigraphy measures for physical activity and for sleep. A sample size of 55 achieves 90% power to detect a correlation coefficient of 0.42 and 80% power to detect a coefficient of 0.37, using a two-sided hypothesis test with a significance level of 0.05. Multiple comparison adjustments will be made to protect the false discovery rate.

### Statistical analyses

#### Primary analyses

We intend to use an intention-to-treat (ITT) approach in all analyses; that is, the assessment of individuals will be analyzed by randomized group, regardless of participation in any classes. This approach will protect the effects of randomization from confounding introduced by subject dropout and crossover.

Compared to per protocol (PP) analysis, ITT is also considered conservative in the context of superiority hypothesis testing (Hypotheses 1 and 2, which are used to power this study). A main analysis will be performed of all valid observed data under a plausible assumption about the missing data. This will be followed by sensitivity analyses that accounts for all randomized patients, to explore the effect of departures from the assumption made in the main analysis. In addition, we note that in the context of non-inferiority hypothesis testing (Hypothesis 1c), the use of ITT instead of being conservative may bias the results toward non-inferiority. We will, thus, for Hypothesis 1c perform PP analysis as well as ITT analysis. Non-inferiority will be achieved only if both analyses afford the same inference of non-inferiority.

In our comparisons of treatments based on the outcome measures, we will analyze outcomes assessment using a number of statistical methods. For instance, for our primary hypotheses, a two-sample t-test will be used to test the mean difference in the SPP group compared with the HE, the mean difference in the eight-week CBT group compared to the HE group, and the mean difference in the SPP group compared to the eight-week CBT group. An rmANOVA will be used to test whether SPP will be superior to HE and non-inferior to eight-week pain-CBT for longitudinal changes in PROMIS pain intensity and behavior, fatigue, and sleep disturbance. Finally, the association between changes in PC provided by SPP and objective physical function will be tested with correlation coefficients between changes from baseline to primary endpoint in PC and in each of the actigraphy measures for physical activity and for sleep.

#### Secondary objectives

Multi-level modeling (MLM) will be used to characterize treatment effects on Daily PC fluctuations, and the impact of Daily PCS changes on Trait PC, longitudinal PROMIS measures, actigraphy (activity and sleep). For example, MLM will be used to account for subject-level (level 2) effects, particularly the effect of the intervention as well as daily level variations in PC. In addition, MLM will be used to test whether the relationship between daily PC and levels in same-day and next-day pain and activity is reduced by SPP and CBT interventions compared to the HE group, using the intervention as the moderator of Daily PC effect on next-day pain and activity. An analysis of level-2 variance will be performed in the MLM framework to test whether Daily PC mean changes from baseline to one month post treatment will predict mean change in pain, activity, sleep, and Trait PC from one to three months post treatment (for the SPP and CBT groups). Finally, MLM will be used to test whether previous-day use of SPP / CBT skills will predict next day improvement in Daily PC. The analysis will account for subject-level (Level 2) effects, particularly daily level variations in use of SPP skills.

## Discussion

In this trial, we will seek to determine whether a single-session targeted PC class is an effective treatment option for persons with chronic back pain. The efficient format of SPP will facilitate application across a variety of settings, such as in primary care or in pre-surgical populations where PC treatment stands to prevent the development of CLBP [7, 25, 35]*.* The SPP intervention is highly scalable and easily lends itself to broad dissemination across settings—outpatient, inpatient, pre-surgical, and community through various technologies (e.g. DVD or a web-based video class). In this trial, we will compare the effectiveness of a single-session PC class with that of CBT, which has been found to be effective for back pain but is not widely available. The study will also characterize how reduced variability in Daily PC and mean levels of Daily PC influence future Trait PC, thereby revealing the mechanisms that facilitate enduring changes in PC. Such critical knowledge will facilitate the development of interventions that specifically target the factors that facilitate positive cognitive and emotional neuroplasticity. Additional validation studies will be required before implementation in other patient populations. If the single-session PC class is shown to be an effective treatment for patients with CLBP, with its low cost and low burden, this treatment could dismantle many of the current barriers and burdens of effective pain care Additional file 1.

### Trial status

Protocol (NCT03167086) was registered on 22 May 2017 and recruitment began in June 2017. The first participant was enrolled in the study on 8 June 2017. Recruitment will be completed in May 2020.

## Additional file

Additional file 1: SPIRIT 2013 Checklist. (DOC 123 kb)
